# Marker Assisted Gene Pyramiding (MAGP) for bacterial blight and blast resistance into mega rice variety “Tellahamsa”

**DOI:** 10.1371/journal.pone.0234088

**Published:** 2020-06-19

**Authors:** Md. Jamaloddin, Ch. V. Durga Rani, G. Swathi, Ch. Anuradha, S. Vanisri, C. P. D. Rajan, S. Krishnam Raju, V. Bhuvaneshwari, R. Jagadeeswar, G. S. Laha, M. S. Prasad, P. V. Satyanarayana, C. Cheralu, G. Rajani, E. Ramprasad, P. Sravanthi, N. Arun Prem Kumar, K. Aruna Kumari, K. N. Yamini, D. Mahesh, D. Sanjeev Rao, R. M. Sundaram, M. Sheshu Madhav

**Affiliations:** 1 Institute of Biotechnology (IBT), PJTSAU, Hyderabad, India; 2 International Rice Research Institute (IRRI), Los Banos, Philippines; 3 Agricultural Research Station (ARS), ANGRAU, Nellore, India; 4 Andhra Pradesh Rice Research Institute (APRRI), ANGRAU, West Godavari, India; 5 Agricultural Research Institute (ARI), PJTSAU, Hyderabad, India; 6 Indian Institute of Rice Research (ICAR-IIRR), Hyderabad, India; Louisiana State University, UNITED STATES

## Abstract

Bacterial blight (BB) and fungal blast diseases are the major biotic constraints that limit rice productivity. To sustain yield improvement in rice, it is necessary to developed yield potential of the rice varieties by incorporation of biotic stress resistance genes. Tellahamsa is a well-adapted popular high yielding rice variety in Telangana state, India. However, the variety is highly susceptible to BB and blast. In this study, simultaneous stepwise transfer of genes through marker-assisted backcross breeding (MABB) strategy was used to introgress two major BB (*Xa21* and *xa13*) and two major blast resistance genes (*Pi54* and *Pi1*) into Tellahamsa. In each generation (from F_1_ to ICF_3_) foreground selection was done using gene-specific markers viz., pTA248 (*Xa21*), *xa13prom* (*xa13*), *Pi54MAS* (*Pi54*) and RM224 (*Pi1*). Two independent BC_2_F_1_ lines of Tellahamsa/ISM (Cross-I) and Tellahamsa/NLR145 (Cross-II) possessing 92% and 94% recurrent parent genome (RPG) respectively were intercrossed to develop ICF1—ICF_3_ generations. These gene pyramided lines were evaluated for key agro-morphological traits, quality, and resistance against blast at three different hotspot locations as well as BB at two locations. Two ICF_3_ gene pyramided lines viz., TH-625-159 and TH-625-491 possessing four genes exhibited a high level of resistance to BB and blast. In the future, these improved Tellahamsa lines could be developed as mega varieties for different agro-climatic zones and also as potential donors for different pre-breeding rice research.

## Introduction

In the current scenario, paddy cultivation facing major threats by a few of biotic stresses throughout the South Asia and ASEAN countries, including India [1]. The intensity of biotic stress in rice production is escalating rapidly at an alarming rate in recent times due to climate change [2]. To meet the demand, rice production new rice varieties possessing strong resistance against biotic factors or by incorporating the multiple resistance (*R*) genes in the local popular high yielding rice varieties may help in food sustainability [3]. However, pyramiding multiple resistance genes through conventional breeding methods is cumbersome due to the dominance and epistatic effects and linkage drag of genes governing such resistance [4]. Marker-assisted selection (MAS) offers a unique advantage to generate pyramided lines that can offer durable resistance in a straight manner by overcoming the limitations of conventional breeding. The availability of linked markers is a valuable resource in marker-assisted backcross breeding (MABB) to pyramid various disease resistance genes. Several biotic stress resistant rice cultivars have been successfully developed with the application of marker-assisted selection [5]. Among the biotic stresses, Bacterial Blight (BB) and fungal blast are the most devastating diseases causing significant yield loss in rice production. Bacterial blight caused by *Xanthomonas oryzae pv*. *oryzae (Xoo)* reduces rice yield drastically by declining photosynthetic area [6]. Disease survey data from the last 34 years within rice cultivating agroclimatic regions of India indicated that BB geographically spread rapidly, as a result of which, in recent years the extent of yield loss due to BB is more than 50% [7]. None of the several tested chemicals or antibiotics could control the BB infestation completely [8]. Therefore, the development and deployment of BB resistant rice varieties would be the most effective and sustainable approach. So far, more than 42 BB resistance genes have been identified; out of which 9 BB genes have been cloned [9]. In Indian environmental conditions, the combination of *Xa21+ xa13* has been known to be more effective against the most virulent races existing across different agro-climatic zones [7].

Whereas rice blast disease caused by fungus *Magnaporthe oryzae* (Mo) is another most important fungal diseases [10]. Previous studies showed about 85 countries across the world are facing serious problems due to rice blast [11]. Estimated yield loss due to blast can be more than 50% when the disease occurs in epidemic proportions [12] and severe cases, it can reach up to 60–100% [13]. Till now, more than 100 distinctive blast resistance genes have been identified [14] and out of them, 21 genes have been cloned [15]. Two dominant resistance genes (*Pi54* and *Pi1*) are known to confer resistance against the major virulent races of the pathogen in India and widely used by different research groups [16]. Enormous genetic diversity exists among the BB and blast pathogens across the geographical regions [17], and previous studies indicate that cultivars with a single resistance gene do not provide broad-spectrum resistance [1]. Therefore, pyramiding of multiple BB and blast resistance genes into high yielding rice varieties through marker-assisted backcross breeding (MABB) maybe the most effective approach to develop durable resistance in rice varieties.

Rice production in the dry season (*Rabi*) is also affected by abiotic stresses like the low temperature at the seedling stage and high temperature at the panicle initiation stage. Tellahamsa, an early maturing (110 days) and cold tolerant variety was released in 1968. It is a mega variety for *Rabi* season and has maximum area under cultivation in Telangana (http://www.rkmp.co.in) due to its locally acceptable cooking quality. However, this variety is highly susceptible to BB and blast. Hence, the improvement of Tellahamsa by pyramiding effective resistance genes of BB and blast can help this variety to remain in breeders’ chain for several years. Few research groups in India have successfully introgressed one or two resistance genes either of BB or blast independently into popular varieties like Pusa Basmati, Samba Mahsuri, PR106, and MTU1010 [18–21], but there were no efforts made to improve Tellahamsa. Considering all these points, the present study is planned to pyramid *Xa21*, *xa13*, *Pi54*, and *Pi1* genes simultaneously in the background of Tellahamsa.

## Material and methods

### Plant material

A well-adapted popular *Rabi* season cold-tolerant rice variety, Tellahamsa (C10754; Parentage: HR 12 x TN-1) released in 1968 from Acharya NG Ranga Agricultural University (ANGRAU), Rajendranagar, Hyderabad was chosen as the recurrent parent. Improved Samba Mahsuri (ISM, having *Xa21* and *xa13*) [19] and NLR 145 (Swarnamukhi, having *Pi54* and *Pi1*) [22] were chosen as donor parents. Both the donors collected from the Indian Institute of Rice Research (IIRR), Hyderabad, Telangana, India.

### Molecular marker analysis

#### Foreground selection

A parental polymorphism survey for target BB genes *Xa21* and *xa13* was conducted using *pTA248* [23] and a functional marker *xa13* Prom [19] respectively between Tellahamsa and ISM. Similarly, for *Pi54* and *Pi1*, *a* functional marker *Pi54MAS* [24] and RM 224 [25], were used respectively **(S1 Table).**

#### Background selection

To identify plants with maximum recurrent parent genome recovery, a set of 565 SSR markers spread across the entire rice genome were used to check polymorphism among the recurrent and donor parents [21]. The primer sequences of the SSR markers were acquired from Gramene SSR marker resources (www.gramene.org). Recurrent parent genome (RPG) recovery was estimated using polymorphic SSR markers with the help of Graphical Genotype (GGT) Version 2.0 [26] software.

### Crossing scheme and MABB for introgression of BB and blast genes into Tellahamsa

**Fig 1:**
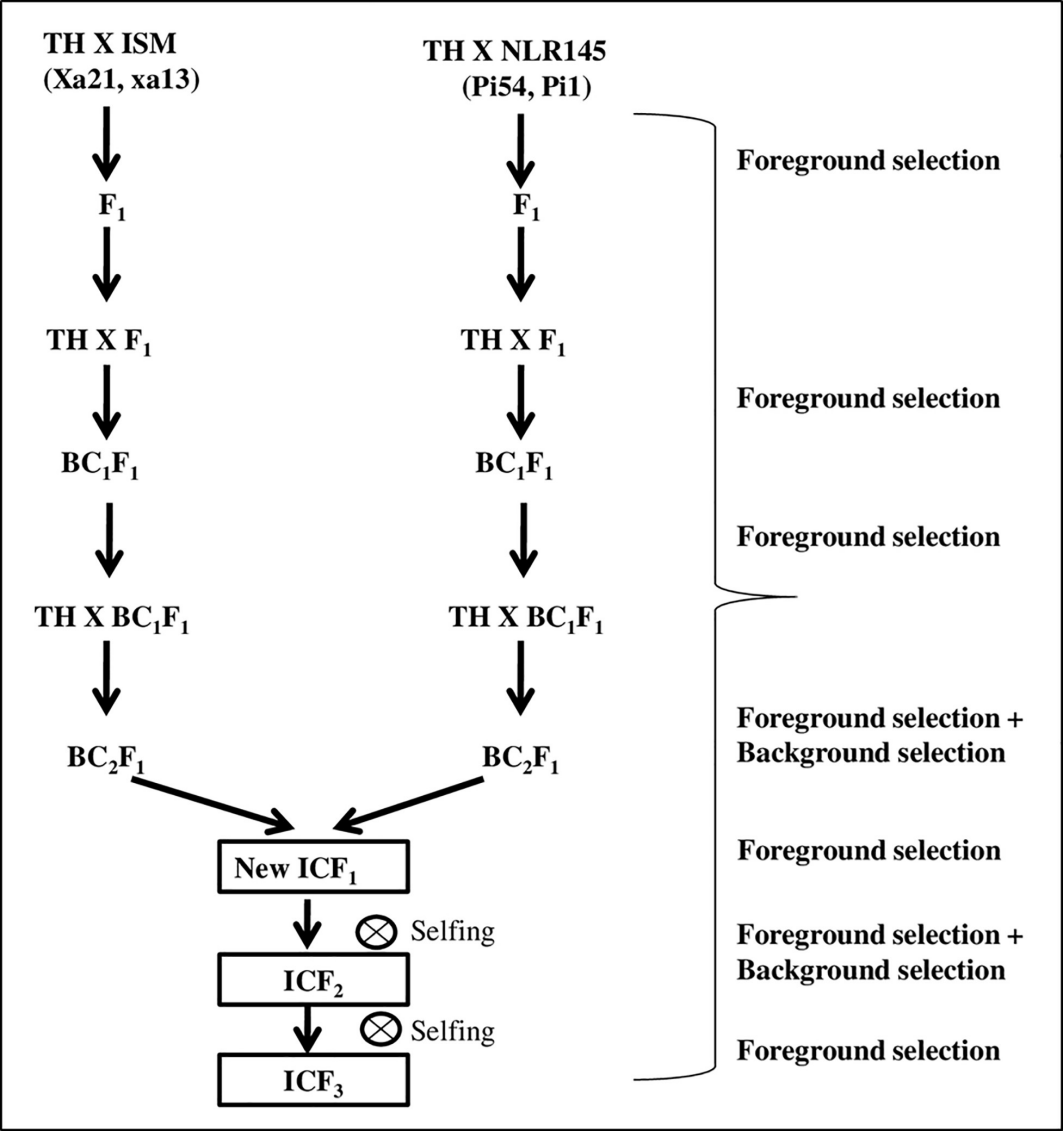
Marker assisted backcrossing scheme adopted in the study.

Simultaneous step wise back cross transfer approach was used to introgress all the four genes into one genetic background [35]. Two independent back-crossing programs, Tellahamsa × ISM (Cross-I) and Tellahamsa × NLR145 (Cross-II) were started for the development of resistant lines using Tellahamsa as the female and two donor parents (ISM and NLR145) as males. The methodology of marker assisted backcross breeding (MABB) strategy adopted in the study is depicted in **Fig 1**. The “true” F_1_ hybrids from both crosses were identified with foreground markers and were backcrossed with Tellahamsa to generate BC_1_F_1_s_,_ that were confirmed for the presence of resistance genes i.e., *Xa21* and *xa13* (Cross I) and *Pi54* and *Pi1* (Cross II) in heterozygous condition. Positive BC_1_F_1_ lines were again backcrossed with Tellahamsa to generate BC_2_F_1_s_,_ which were then screened with foreground molecular markers as described earlier. Two independent BC_2_F_1_ lines from each cross possessing *Xa21* and *xa13* (Cross I) and *Pi54* and *Pi1* (Cross II) in heterozygous condition with maximum recovery of Tellahamsa genome were identified with the help of a set of polymorphic SSR markers. Those lines were intercrossed to generate intercross F_1_s (ICF_1_) to combine the resistance genes *Xa21 + xa13 +Pi54 + Pi1* into a single plant. ‘True’ ICF_1_ hybrids were identified after screening with the resistance genes specific/linked markers (foreground markers) and selfed for two generations to generate ICF_3_.

### DNA isolation and PCR amplification

Genomic DNA was isolated from the leaf samples by following a standard protocol used for plants [27]. The isolated DNA samples were checked for their quality and quantity by using agarose gel electrophoresis (0.8% agarose gel) and spectrophotometer (Thermo electronic corporation UV1) respectively and then were used to set the PCR reactions [35]. The PCR reaction mixture contained 50ng template DNA, 5 picoM each of forward and reverse primers, 200μM dNTPs, 1X PCR buffer (10mM Tris-HCl, pH 8.3, 50mM KCl, 1.5mM MgCl_2_ and 0.01mg/ml gelatin) and 0.5U of *Taq* DNA polymerase (JONAKI) in a reaction volume of 10μl. Amplification cycling was performed in a gradient programmable master cycler (Veriti, Applied Biosystems). The PCR condition was with one cycle of denaturation at 95°C for 5 min, followed by 35 cycles at 95°C for 45s, 55°C for 45s, and 72°C for 1 min, and with a final extension at 72°C for 10 minutes. The final PCR products were resolved by electrophoresis on 3% agarose (Sea Kem, Lonza) gel and documentation was done using gel documentation system (BIO- RAD), images were stored for further scoring and permanent records.

### Screening for BB resistance

The selected nine ICF_2_ lines carrying BB resistance genes along with parents were transplanted into the main field with a spacing of 15 x 20 cm and inoculated with a bacterial suspension culture of 10^8-9^cfu/ml containing two most virulent isolates of *Xanthomonas oryzae pv*. *Oryzae* (DX-020 from Hyderabad, Telangana and IC-31 from Maruteru, Andhra Pradesh), at maximum tillering stage at two different locations (Agricultural Research Institute (ARI), Hyderabad, India and Andhra Pradesh Rice Research Institute (APRRI), Maruteru, India) during *Rabi* 2014–15. The leaf clipping method of inoculation was used to inoculate five young leaves in each plant [28] and the disease reaction was recorded 21 days after inoculation both by visual scoring and measurement of lesion length (LL) as per standard evaluation system (SES) scale of IRRI, 1996 [29].

### Screening for blast resistance

Artificial screening for rice blast was carried out in nine ICF_2_ lines along with parents during *Rabi*, 2014–15 with three highly virulent isolates of *Magnaporthe oryzae* (IB-16 (Maruteru), ID-14 (Nellore) and NLR-1 (Hyderabad) at three key locations of Andhra Pradesh and Telangana states (Andhra Pradesh Rice Research Institute (APRRI), Maruteru, India, Regional Agricultural Research Station (RARS), Nellore, India and Agricultural Research Institute (ARI), Hyderabad, India) in Uniform Blast Nursery (UBN). Each test entry was sown in a single row of 50 cm length and successive rows were 10 cm apart. After every 10 test entries and around the nursery the susceptible check (HR12) was sown for uniform spread of blast disease to the experimental material. The pathogen strains mentioned above were cultured and stored as described in the standard protocol [30]. The young seedlings at four-leaf stage were inoculated with the fungal conidial suspension at a concentration of 1 × 10^5^ spores/ml and high relative humidity was maintained for the disease development. Inoculated seedlings were monitored for the development of blast lesions one week after inoculation and the plants were scored on a 0–9 scale as per IRRI-SES (IRRI, 1996) [29].

### Evaluation of agronomic performance and grain quality analysis

Thirty days old seedlings of selected ICF_2_ lines and parents were transplanted into the main field with a spacing of 15 x 20 cm at Agricultural Research Institute (ARI), Hyderabad, India. Standard agronomic practices were followed to raise a healthy crop and the lines were evaluated during the *Rabi* season, 2014–15. Data of various agro-morphological traits viz., plant height (PH), number of productive tillers per plant (NT), days to 50% flowering (DFF), panicle length (PL), number of grains per panicle (GN), yield per plant (GY), 1000 grain weight (GW) and grain type (GT) were recorded. Grain quality traits were analyzed in three replications to record grain size (GS), Kernel Length (KL), Kernel Length After Cooking (KLAC), Kernel Breadth (KB), Kernel Breadth After Cooking (KBAC), Elongation Ratio (ER), Amylose Content (AC), Alkali Spreading Value (ASV) and Gel Consistency (GC). Kernel length and kernel breadth were measured by dial micrometer and length breadth ratio was calculated Amylose content was determined by the relative absorbency of starch iodine color in a digested solution of 100-mesh rice flour by Juliano’s modified method [31]. Gel consistency, which classifies the quality of cooked rice, was measured by following a described method [32].

### Data analysis

The marker data was analyzed using the Graphical Genotype (GGT) Version 2.0 software [21] package to estimate the percentage of recurrent parent chromosomal segment recovery in the selected segregants of the backcrossed population. Analysis of variance (ANOVA) of the replicated agronomic data was performed using GenStat (http://www.biosci.global/softwar-en/genstat/) statistics software.

## Results

To improve the cold tolerant early maturing rice variety, Tellahamsa for BB and blast disease resistance, Marker-Assisted Back Crossing (MABC) coupled with stringent phenotypic screening was employed. The introgressed ICF_3_ lines of Tellahamsa, namely, TH-625-159 and TH-625-491 (carrying two BB resistance genes (*Xa21*and *xa13)* and two blast resistance genes (*Pi54* and *Pi1)* were evaluated under current study.

### Introgression of BB resistance genes (*Xa21* and *xa13*) into Tellahamsa (Cross-I)

60 F_1_ hybrids were generated by crossing Tellahamsa x ISM (Cross-I) and were screened for their heterozygosity using the gene specific foreground markers, pTA248 for *Xa21* and *xa13 prom* for *xa13*. The selected heterozygous F_1_ hybrids were backcrossed to recurrent parent to generate 202 BC_1_F_1_ lines, which were again confirmed with the same gene specific foreground markers. Depending on the agro-morphological resemblance to the recurrent parent, a total of 22 heterozygous BC_1_F_1_ lines for *Xa21* and *xa13* genes were selected and used to generate BC_2_F_1_ lines. A total of 102 BC_2_F_1_ lines were produced, of which 6 BC_2_F_1_ lines showed the presence of *Xa21* and *xa13* genes in heterozygous condition. All these plants were subjected to back ground selection with sixty polymorphic SSR markers and they exhibited presence of 88% to 94% of recurrent parent genome with an average of 89.3%. Out of which 6 lines, 2 BC_2_F_1_ lines (TH- BC_2_F_1_-16 and #TH- BC_2_F_1_-196) possessing maximum RPG recovery (91% and 94% respectively) were identified and used for intercrossing.

### Introgression of blast resistance genes (*Pi54* and *Pi1*) into Tellahamsa (Cross-II)

A total of 125 F_1_ hybrids were produced by crossing Tellahamsa x NLR145 (Cross-II) and 52 hybrids were observed to be “true” F_1_s based on PCR results with gene specific marker *Pi54MAS* for *Pi54* and gene linked marker RM 224 for *Pi1*. F_1_ hybrids were backcrossed to recurrent parent to generate 200 BC_1_F_1_ lines. Depending on agro-morphological resemblance to the recurrent parent, a total 28 heterozygous lines for *Pi54* and *Pi1* genes were selected and used to generate 152 BC_2_F_1_ lines. Out of 152 BC_2_F_1_ lines, 13 BC_2_F_1_ lines showed the presence of *Pi54* and *Pi1* in heterozygous condition and were subjected to back ground selection with sixty polymorphic SSR markers. The current study found 88% to 94% recurrent parent genome recovery with an average of 89.7%. Out of 13 lines, 2 lines (TH- BC_2_F_1_-6 and #TH- BC_2_F_1_-105) possessing maximum RPG recovery (93% and 94% respectively) and good phenotypic similarity to recurrent parent Tellahamsa were identified and used for inter- crossing.

### Pyramiding of BB and blast resistance genes

The line TH- BC_2_F_1_-196 (derived from cross-I) was used as female parent and TH- BC_2_F_1_-105 (derived from cross-II) as male parent in the intercross to pyramid *Xa21*, *xa13*, *Pi54* and *Pi1* genes. Out of 152 ICF_1_ lines, 3 lines were confirmed to be heterozygous for all the target genes (i.e., *Xa21+ xa13 + Pi54* + *Pi1*). Among these, one intercross F_1_ hybrid (20th hybrid) was selected based on DFF (days to 50% flowering)_,_ plant height and grain type characters as like Tellahamsa and further it was selfed to obtain 1012 ICF_2_ lines. Foreground selection was carried out in these 1012 ICF_2_ lines for identifying the lines carrying a combination of two, three and four genes using the respective foreground markers **(S1 Fig)**. Based on foreground and phenotypic selection for agro-morphological characters, a total of 9 homozygous lines with different gene combinations were identified. Four lines with *Xa21 + xa13 + Pi54 + Pi1*, three lines with *Xa21 + xa13 + Pi54*, one plant with *Xa21+Pi54 + Pi1* and one plant with *Xa21 + xa13 + Pi1* gene combination were identified. Background analysis was carried in those selected 9 lines with remaining polymorphic SSR markers which were still heterozygous in BC_2_F_1_ generation. Two lines TH-625-159 (94.8%) and TH-625-491 (95.6%) were identified based on highest recurrent parent genome recovery and acceptable phenotypic characters **(S2 Table)**.

### Agro-morphological and quality characters of selected ICF_2_ pyramid lines

The agro-morphological data of nine selected ICF_2_ lines indicated that the plant height of the lines ranged from 96.5 cm (ICF_2_-TH-625-491) to 101 cm (ICF_2_-TH-625-105 and ICF_2_-TH-625-159), while, the recurrent parent (Tellahamsa) was about 95 cm tall. Most of the recombinants showed a plant height nearly equal to the recurrent parent. The mean panicle bearing tiller (PBT) number varied from 8.0 to 14.0 and some lines showed slightly lower tiller number than the recurrent parent (13.0) and two lines (ICF_2_-TH-625-159 and ICF_2_-TH-625-325) showed equal number of tillers as that of recurrent parent. Panicle length (PL) of one line, ICF_2_-TH-625-159 was found to be on par with recurrent parent Tellahamsa (26.5 cm), while in all others the values were nearer to or slightly lower than that of the Tellahamsa. The 1000-grain weight was higher in TH-625-159 (25.00 g) and lowest in ICF_2_-TH-625-501 (18.7 g) (**S2 Table**).

The grain cooking qualities of selected lines were observed close to recurrent parent (Tellahamsa) in context to background selection. The analysis of variance showed highly significant value for all the traits undue study **(Table 1).** Kernel length of the introgression lines varied from 5.26 cm (ICF_2_-TH-625-588) to 6.46 cm (ICF_2_-TH-625- 21), the kernel breadth varied from 1.76 (ICF_2_-TH-625-491) to 2.10 (ICF_2_-TH-625-588) and the L/B ratio ranged between 2.50–3.35, while the recurrent parent Tellahamsa had an L/B ratio of 2.90. The ASV value for, ICF_2_-TH-625-211 and ICF_2_-TH-625-325 lines were recorded as similar values like Tellahamsa (6), while the remaining lines had a slightly higher value (7). The KLAC of the lines were ranged from 8.43 to 9.97 mm but few lines were found to be better than Tellahamsa (9.43 mm). All the Improved lines had amylose content in the range of 7.9–33.5% and the lines viz; ICF_2_-TH-625-159, ICF_2_-TH-625-491 and ICF_2_-TH-625-624 recorded values nearer to that of Tellahamsa (19.2%).

**Table 1:** Cooking quality attributes of improved lines.

S.NO	Entries	KL	KB	L/B ratio	KLAC	KBAC	ER	ASV	GC	AC
1	ICF_2_-TH-625- 21	6.46	1.93	3.35	9.90	2.93	1.53	7	60	22.4
2	ICF_2_-TH-625-105	5.83	2.00	2.92	9.97	2.77	1.71	7	55	10.8
3	ICF_2_-TH-625-159	5.83	1.96	2.97	9.37	2.70	1.61	7	30	21.4
4	ICF_2_-TH-625-211	5.83	1.93	3.02	8.43	2.83	1.45	6	30	33.5
5	ICF_2_-TH-625-325	5.83	1.96	2.97	8.87	2.90	1.52	6	35	11.6
6	ICF_2_-TH-625-491	5.63	1.76	3.20	9.23	2.20	1.64	7	45	18.3
7	ICF_2_-TH-625-501	5.96	2.00	2.98	9.27	3.03	1.55	1	50	7.9
8	ICF_2_-TH-625-588	5.26	2.10	2.50	8.77	2.60	1.67	7	30	33.8
9	ICF_2_-TH-625-624	5.40	1.93	2.80	9.30	2.87	1.72	7	30	18.5
10	Tellahamsa	5.80	2.00	2.90	9.43	2.93	1.63	6	35	19.2
11	NLR145	5.90	2.00	2.95	9.30	2.90	1.58	2	45	23.3
12	ISM	5.00	1.56	3.21	8.67	2.67	1.73	1	40	21.5

KL = kernel length, KB = kernel breadth, L/B ratio = length/ breadth ratio, KLAC = kernel length after cooking, KBAC = kernel breadth after cooking, ER = elongation ratio, ASV = alkali spreading value, GC = gel consistency and AC = amylose content.

### Screening of ICF_2_ lines for blast and BB resistance

Artificial screening for rice blast was carried out in nine ICF_2_ lines during *Rabi*, 2014–15, against high virulent local isolates of blast pathotypes (IB-16 (Maruteru), ID-14 (Nellore) and NLR-1 (Hyderabad) at three locations and for BB (DX-020 from Hyderabad, Telangana and IC-31 from Maruteru, Andhra Pradesh) in two locations. Among the nine ICF_2_ lines, two lines #TH-625-159 and #TH-625-491 showed small brown specks of pinhead size without a sporulating center on the leaves and recorded a mean blast disease score of 1.0 and 1.6 respectively (**S2A, S2B and S2C Fig; S3 Table)**. Similarly, these two lines also showed BB resistance with a score of 1.0 (**S2D and S2E Fig; S3 Table).**

### Recombinant selection

**Fig 2:**
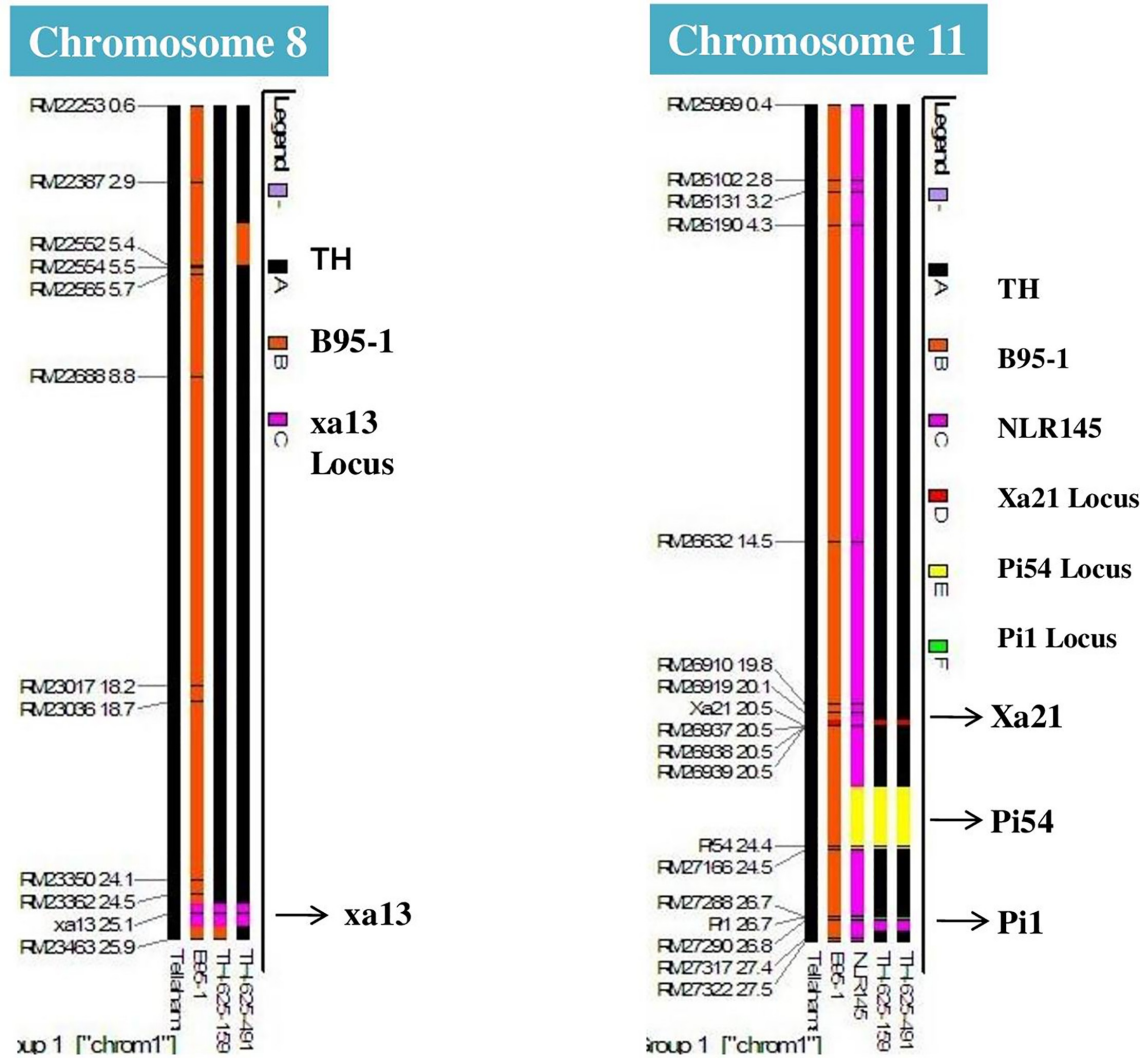
Representation of graphical genotype of selected ICF_2_ lines in the genomic region around, *xa13* on Chromosome- 8 based on analysis with parental polymorphic SSR markers. A- Tellahamsa -The recurrent parent Tellahamsa (represented by black color), B- ISM- donor parent for *xa13*, (represented by Orange color) introgression of *xa13* locus in the best two ICF_2_ lines (represented by dark lavender color).

To estimate the extent of “linkage drag” around the four target genes, viz., *xa13* (Chromosome 8), *Xa21*, *Pi54* and *Pi1* (Chromosome 11), two lines (TH-625-159, and TH-625-491) were subjected to analysis with markers. In the case of *xa13*, a segment of 0.2 Mb was observed to have gotten introgressed at the proximal end from the donor parent genome, while at the distal end, a segment of 0.3 Mb was observed to have gotten introgressed, thus limited to ∼0.5 Mb segment that was transferred from the donor parent. In case of *Xa21*, a segment of 0.3 Mb at the proximal end and 0.2 Mb at the distal end were observed to have gotten introgressed. With respect to *Pi54*, segments of 3.0 and 0.5 Mb (totaling to 3.5 Mb) and with respect to *Pi1*, segments of 0.2 and 0.3 Mb (totaling to 0.5 Mb) had gotten introgressed from the donor parent genome **(Fig 2)**. The best ICF_2_ lines (i.e., # TH-625-159 and TH-625-491) were forwarded to next generation, ICF_3_ by selfing.

Representation of graphical genotype of a selected ICF_2_ lines in the genomic region around *Xa21*, *Pi54 and Pi1* on Chromosome—11. Tellahamsa -The recurrent parent Tellahamsa (represented by black color, i.e. A), (ISM/B95-1), the donor parent for *Xa21*, (represented by Orange color, i.e. B and *Xa21* locus represented by dark red color) and NLR145, the donor parent for *Pi54* and *Pi1 (*represented by dark lavender color, i.e. C and *Pi54* represented by yellow color and *Pi1* represented by ash color).

### Foreground selection of ICF_3_ lines

Two ICF_3_ lines derived from #TH-625-159 (100 lines) and #TH-625-491(100 lines) were found to be homozygous for all the four target resistance genes which were confirmed by respective foreground marker analysis. **(S3 Fig).**

### Evaluation of BB and blast resistance of ICF_3_ Lines

**Fig 3:**
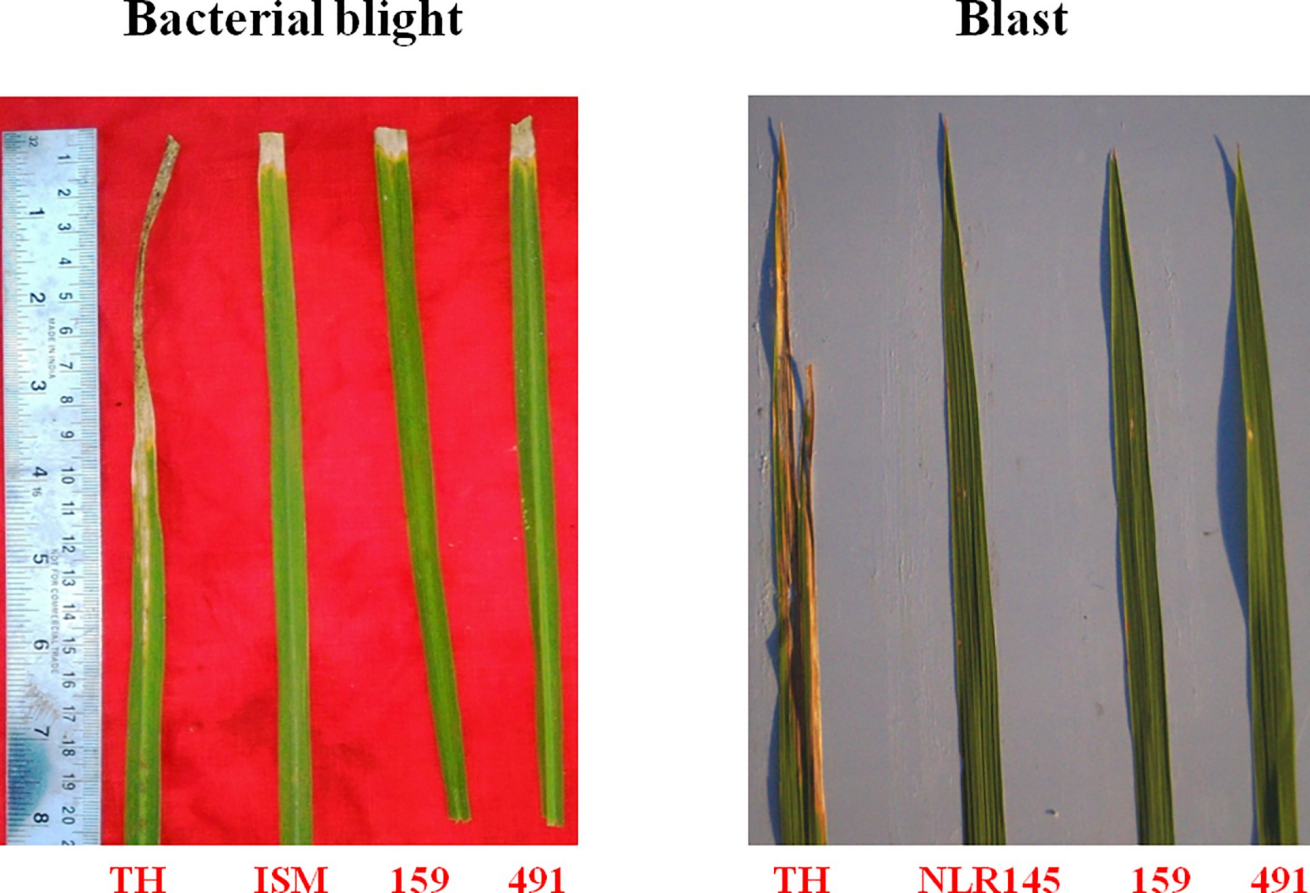
Leaves of ICF_3_ lines along with parents showing symptoms of BB and blast.

**Fig 4:**
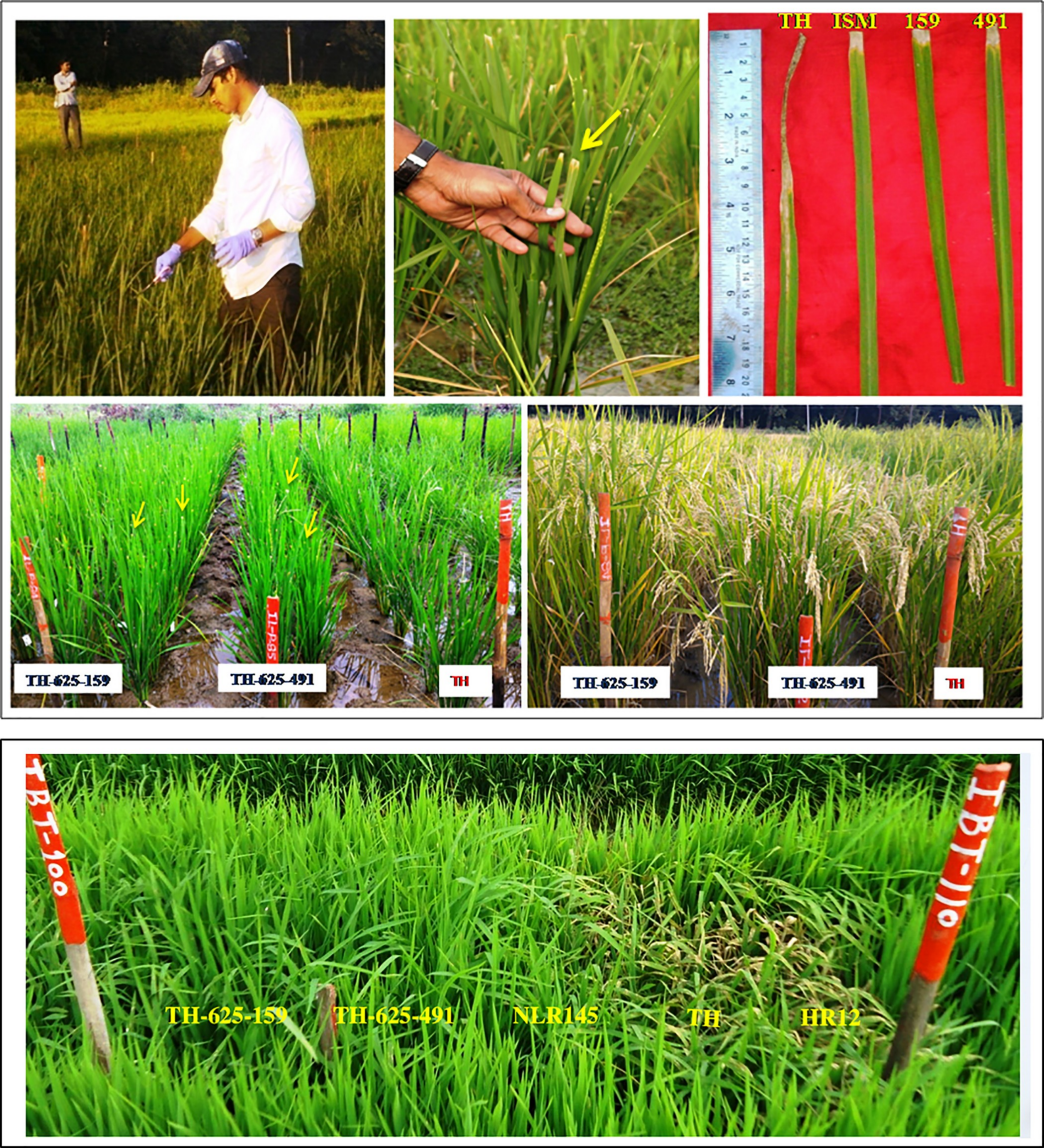
**a:** BB screening of ICF_3_ (TH-625-159 & TH-625-491) lines at ARI, Hyderabad, During *Kharif*, 2015–16. Screening of selected ICF_3_ (TH-625-159 & TH-625-491) lines having *Xa21+xa13+Pi54+Pi1* genes against local BB isolate (DX-020). ICF_3_ progenies were highly resistant against BB isolate (DX-020). TH: Tellahamsa- Recurrent parent (Susceptible); ISM: Improved Samba Mahsuri (Resistant); 159 and 491: ICF_3_progenies (TH-625-159 and TH-625-491). **b:** Blast screening of ICF_3_ (TH-625-159 & TH-625-491) lines at IIRR, Hyderabad, During *Kharif*, 2015–16. Screening of selected ICF_3_ (TH-625-159 & TH-625-491) lines having *Xa21+xa13+Pi54+Pi1* genes against local blast isolate (NLR-1). ICF_3_ lines were highly resistant against blast isolate (NLR-1). TH: Tellahamsa- Recurrent parent (Susceptible); NLR145- (Resistant check); HR12- (Susceptible check); 159 and 491: ICF_3_ lines (TH-625-159 and TH-625-491).

ICF_3_ lines possessing BB and blast resistance genes were screened for BB and blast resistance along with resistant donor parents (ISM, NLR-145) and susceptible recurrent parent (Tellahamsa) using a virulent isolate of *Xoo* (DX-020) for BB and NLR-1 for blast collected from Indian Institute of Rice Research (IIRR), Hyderabad. Screening was done under field conditions for BB and in an UBN (Uniform Blast Nursery) for blast during *Kharif* season 2015–16. Tellahamsa showed highly susceptible reaction to BB, whereas ISM (donor for BB) displayed resistance by showing small lesion (0.95 ± 0.5cm). ICF_3_ lines were showed the BB score in the range of 0.5 to 1.0. While screening for blast disease, NLR-145 showed a score of 1, On the same time, Tellahamsa showed a score of 9.0 and the derived ICF_3_ lines were showed score of 1.0 (**Table 2)**. The pictorial representation of disease scores reflected as symptoms on the leaves has been given in **Fig 3**. The complete process of screening of ICF_3_ lines along with parents for BB and blast has been depicted in **Fig 4A** and **Fig 4B** respectively.

**Table 2:** Agro-morphological characters, disease resistance and amylose content of selected ICF_3_ lines.

Plant identity	(DFF)	PH (cm)	NPP	PL (cm)	GN	GY (g)	1000 seed weight (g.)	GT	BB	Blast	AC
TH-625-159	97.3±0.8	101.5 ± 0.3	14±1.1^*##*^	26.6±0.1	140±1.1^*##*^	27.5±1.3^*##*^	25.0±0.5^*##*^	LS	1^*##*^	1^*##*^	19.80
TH-625-491	90.0±0.5^*#*^	97.7 ±0.4	13±1.5	26.2±0.1	135±1.7^*#*^	28.3±1.8^*#*^	23.8.±0.9^*#*^	LS	1^*#*^	1^*#*^	19.50
Tellahamsa	90.0±0.5	95.1 ±0.8	13.7±0.6	26.6±0.7	130±1.1	25.5±0.6	23.6±0.4	LS	9	9	19.0
B-95-1	95.3±0.3	76.0±1.0	11.7±0.6	22.2±0.5	170±1.1	23.5±0.6	19.7±0.6	MS	2	-	25.20
NLR -145	85.3±0.8	101.8 ±0.9	8.7±0.6	24.1±0.4	184±4.5	28.3±0.5	20.3±0.2	LS	-	1	23.79
CV (%)	1.14	1.21	14.87	1.99	2.96	7.02	5.65				
LCD	1.97	2.14	3.47	0.99	8.46	3.52	2.22				

DFF = Days to 50% flowering; PH = Plant height (cm); NPP = No. of productive panicles/ plant; PL = Panicle length (cm); GN = No. of grains per panicle; GY = Grain yield per plant (g); GT = Grain type; AC = Amylose content

Tellahamsa- Recurrent parent; ISM and NLR145 –Donor parents; MS-Medium slender; LS- Long slender; CV-Coefficient variance; LSD- Least significant difference at 5% probability level; ± -Standard error and values given are mean of three replications; BB and blast score and Amylose content.

# TH-625-491 derived line is better than the recurrent parent in terms of DFF, No. of grain per panicle, Grain yield per plant, 1000 seed weight, both BB and blast resistant.

## TH-625-159 derived line is better than the recurrent parent in terms of No. of productive tillers, No. of grain per panicle, Grain yield per plant, 1000 seed weight, both BB and blast resistant. No. of productive panicles, No. of grain per panicle, Grain yield per plant, 1000 seed weight, both BB and blast resistant

### Evaluation of ICF_3_ lines for yield and morpho-physiological traits

**Fig 5:**
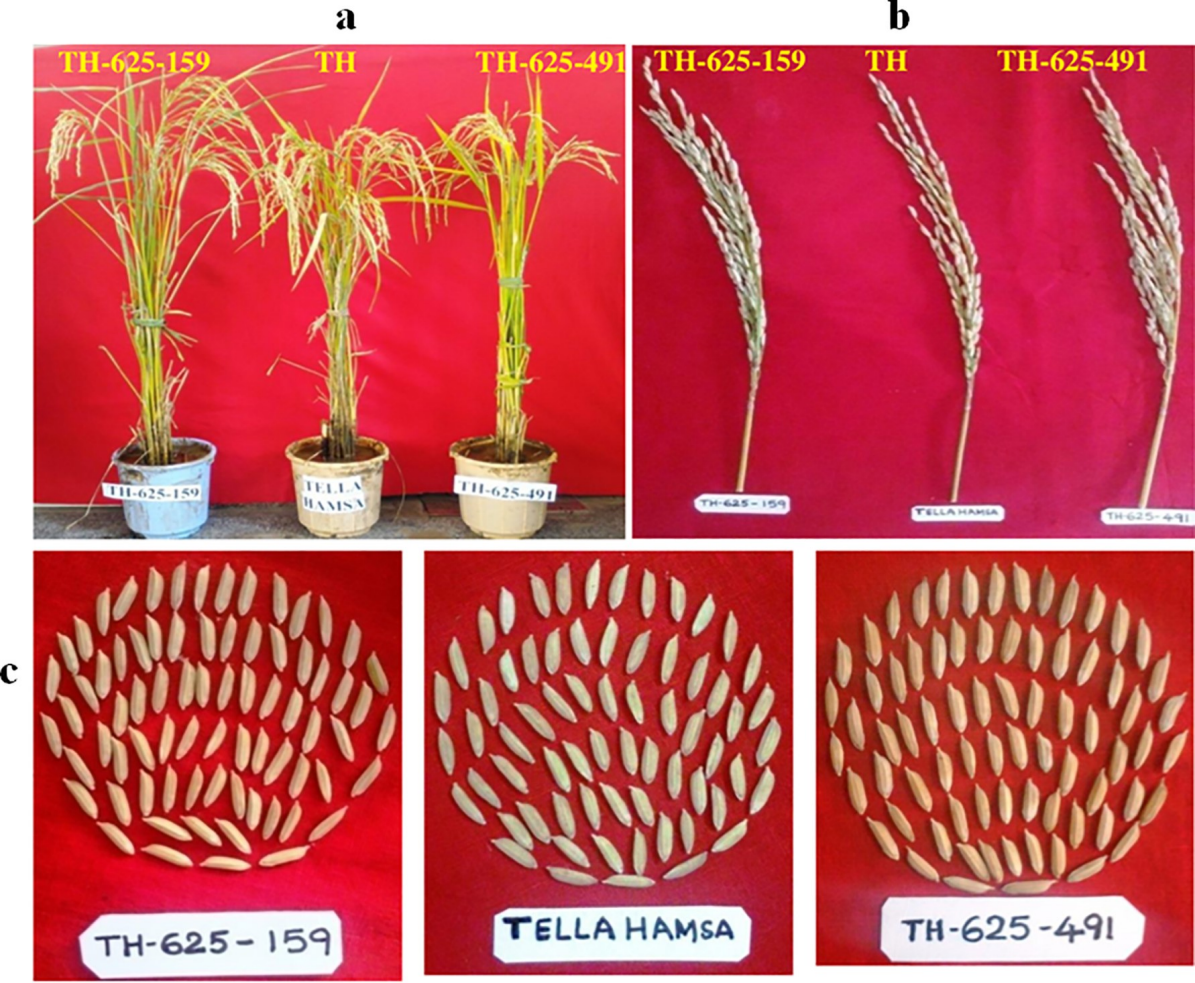
Comparison of agronomic traits such as plant height (5a), grain number per panicle (5b), grain type (5c) of ICF_3_ lines (TH-625-159 and TH-625-491) with recurrent parent Tellahamsa.

The ICF_3_ line derived from #TH-625-491 showed the days to 50% flowering (DFF) as well as plant height data similar to recurrent parent (Tellahamsa) whereas line derived from #TH-625-159 showed a 7 days late in DFF and was taller than recurrent parent **(Fig 5A) (Table 2).** The ICF_3_ line #TH-625-491 was found to be similar to the recurrent parent and had yield advantage over the recurrent parent. However, both the ICF_3_ lines showed higher grain number per panicle (140, 135) as compared to Tellahamsa (130), thereby increase in yield per plant (28.3 gm, 27.5 gm) as compared to recurrent parent (25.5g) **(Fig 5B).** ICF_3_ lines also showed grain type as similarity to the recurrent parent Tellahamsa (Fig 5C). Whereas the grain quality parameters, amylose content in the selected lines TH-625-159 (19.8%) and TH-625-491 (19.5%) was similar to recurrent parent (Tellahamsa) (19.0%) (**Table 2).**

## Discussion

Rice production is always constrained by biotic stresses, among which, bacterial blight (BB) and blast are the important diseases that cause significant yield losses [33]. These diseases are taking an epidemic form due to climate change as a result of which many popular cultivars are becoming susceptible. Fortunately, resistance genes for a wide number of races of BB and blast are available; which can be introgressed to develop the improved version of popular cultivars [34]. The present study demonstrates successful incorporation of genetic resistance to these diseases using MABC in a popular cold tolerant, long slender grain rice variety Tellahamsa. Marker assisted selection is a widely adapted technique which is being used by several researchers to introgress the genes and it becomes more successful when the markers perfectly linked to the trait of interest are available (JGL1798, Jin 23B hybrids and Swarna Sub1) [35–37]. Although there are different resistance sources available for BB, we used ISM (Improved Samba Mahsuri which has *Xa21 xa13* and *xa5*) as donor, since it has good cooking quality and morphological characters and offering a good level of resistance to BB under field conditions in most of the rice growing regions in India. As *xa5* is known to exhibit partial dominance and was reported to display negative effects in gene pyramided lines of some Indian rice varieties [35], in the current study we targeted only two genes (*xa13* and *Xa2*1) out of three genes (*Xa21 xa13* and *xa5)*. ISM has also been used in the earlier molecular breeding studies towards improvement of existing rice varieties [35, 38]. For the introgression of *Pi54* and *Pi1*, in the current study used Swarnamukhi (NLR145), known to have *Pi54 and Pi1* genes, as it was derived from Tetep (CICA-4 × IR-625-23-3-1 × Tetep), while the presence of *Pi1* gene in NLR145 has been reported [22]. Tetep is already being used for introgression of *Pi54* in several molecular breeding studies [39, 38]. NLR145 is much better donor than Tetep, since it has less linkage drag of unwanted morphological traits. In Indian climatic conditions *Pi54*, *Pi1* and *Pi2* offer significant level of resistance to blast across environments [40, 41]. The combinations of *Pi54* and *Pi1* in case of Samba Mahsuri, Swarna, Swarna sub-1 and Pusa Basmati 1 (PB 1) have showed high level of blast resistance [42–45]. Recently, many research workers have reported a highly effective blast resistance of gene pyramided lines possessing *Pi54* and *Pi2* in diverse locations of India [46, 43, 38]. Previous studies also made evident that marker assisted pyramiding of major genes have helped to increase resistance level to BB [47] and blast [25]. Gene specific markers pTA248 for *Xa21*, *xa13 prom* for *xa13*, *Pi54MAS* for *Pi54* and RM224 for *Pi1* were employed for stringent foreground selection. All these markers are highly polymorphic and can be detected very easily so they have great potential to serve as an important tool to introgress *Xa21*, *xa13*, *Pi54* and *Pi1* resistance genes. Although SNPs are ubiquitous and highly abundant in rice genome, keeping in view the high cost of genotyping, we resort to SSR markers for background analysis, which provides a quick evaluation of genetic background among the recombinants. The current study employed simultaneous step wise transfer method for introgression of BB and blast resistance genes.

The two individual BC_2_F_1_ lines having BB and blast genes (cross-I—*Xa21*, *xa13* and cross-II–*Pi54*, *Pi1*) were intercrossed to bring all the genes into a single background. But the current study got lines containing only BB genes and only blast genes in addition to lines having four genes. The current study used high number of parental polymorphic SSR markers (#111) covering the entire genome, particularly focusing on the target chromosomes (i.e., Chr. 8 on which *xa13* gene is located and Chr. 11 on which *Xa21*, *Pi54* and *Pi1* genes are located). The recurrent parent genome recovery of the selected 6 lines (BC_2_F_1_, Cross-I) having two BB genes varied from 88% to 94% with an average of 89.3%. In another cross, the back-ground genome recovery among the 13 lines having *Pi54* and *Pi1* (BC_2_F_1_) varied from 88% to 94% with an average of 89.7%. The process of intensive marker assisted foreground selection at the initial stages coupled with backcrossing resulted in near complete recovery of the recurrent parent genome in two backcrosses with only small segments flanking the target genes possessing donor chromosomal regions. The recurrent parent genome recovery was increased to 95.6% in (ICF_2_) lines as a simultaneous but stepwise backcrossing breeding method was followed [48].

In the present study, nine ICF_2_ lines having combination of four and three genes in homozygous conditions were evaluated for agro-morphological and quality traits, as well as BB and blast resistance. The analysis of variance (ANOVA) showed highly significant value for each character in nine ICF_2_ lines **(Table 3)**. The length/breadth ratio of kernels is very important to determine shape of grains, which is ultimately taken as criteria for fixation of price in the market. The current study identified ICF_2_ lines that are having similar length/breadth ratio as that of Tellahamsa. Elongation ratio is an important parameter for cooked rice [49]. The elongation ratio and amylose contents are also very similar to Tellahamsa (1.63, 19.2%) in selected ICF_2_ lines *viz*., ICF_2_-TH-625-159 (1.61, 21.4%) and ICF_2_-TH-625-491 (1.64, 18.3%). Thus, the MAS derived versions of Tellahamsa (ICF_3_-TH-625-491 and ICF_3_-TH-625-491) can be deployed to replace Tellahamsa, where it is grown especially in *Rabi season* to combat the yield loss due to BB and blast, so sustain the rice productivity.

**Table 3:** ANOVA of variance for five cooking quality characters.

Trait	DF	Sum Sq	Mean Sq	F value	p value
**KL**	11	4.4631	0.4057	117.2774	0*
**KLAC**	11	0.7967	0.0724	15.3369	0*
**KB**	11	7.0675	0.6425	6.479	0.0001*
**KBAC**	11	1.6156	0.1469	3.8038	0.003*
**AC**	11	2326.437	211.4943	91.6238	0*

KL, kernel length, KLAC, kernel length after cooking, KB, kernel breadth, KBAC, kernel breadth after cooking, AC, Amylose content

DF = Degrees of freedom, Sum Sq = sum of square, Mean Sq = Mean sum of square, F Value = Calculated F value and Pr (>F) = F Value with probability

Where * represents significant at 5% probability level, respectively; P = Probability value.

According to earlier reports, development of lines was done by screening at a single location [50]. Screening at multiple locations helps us to identify most stable resistant lines, so the current study screened the ICF_2_ lines for blast resistance at three different locations with different virulent strains to identify the best lines. Two lines (TH-625-159 and TH-625-491) which were similar to Tellahamsa in agro- morphological traits and with good cooking quality characters had advanced for BB and blast screening under controlled conditions by artificially inoculating the virulent isolates of pathogen. These improved lines showed a significantly higher level of BB and blast resistance compared to the recurrent parent Tellahamsa. During previous studies, a high level of resistance to majority of pathotypes of BB by *Xa21* gene was reported in India [51]. However, to enhance the durability of resistance, along with *Xa21*, the current study has additionally introgressed *xa13*. As per previous studies, instead of single gene introgression, multiple genes were showing high level of durable resistance against BB [52]. The data from All India Coordinated Rice Improvement project (AICRIP) (DRR Progress report, Vol. 2, 2008–2013) clearly indicated that NILs of Samba Mahsuri and Swarna possessing *Pi54* gene have high level of resistance across different rice cultivating agro-climatic zones of India. However, in order to enhance the spectrum and durability of blast resistance, along with *Pi54* we introgressed *Pi1* into Tellahamsa. The developed line i.e., #TH-625-491 showed maximum recovery (RPG 96.5%) of the recurrent parent genome and it was more similar to the recurrent parent for different morphological traits viz., (DFF, plant height and effective number of tillers) followed by another line i.e., #TH-625-159 which was having an RPG recovery of 94.8%. The line #TH-625-159 showed a 7 days late in flowering and was slightly taller in plant height (101 cm) as compared to Tellahamsa. Whereas, both the lines showed yield advantage (28.3gm and 27.5 gm) over the recurrent parent (25.5 gm) due to a higher grain number per panicle. Among the grain quality parameters, amylose content is the most important trait, hence the current study analyzed the amylose content in improved derived lines of ICF_3_. In general, amylose content observed within the range between 15% to 35% in rice, [53] while rice varieties with intermediate amylose content (20% - 25%) are more preferred by consumers for consumption around the world since this kind of cooked rice is soft and flaky [54]. In the current study, in ICF_3_ lines #TH-625-159 and #TH-625-491 showed significantly similar level of amylose content (> 20%) as that of the recurrent parent (Tellahamsa). #TH-625-491 line was tested in AICRIP trails in the year 2017 with code number IBT R5 (IET27276) and it showed significant resistance against BB and blast in different test locations with good agro-morphological characters as compared with the recurrent parent Tellahamsa (http://www.icariirr.org/AICRIP/varietal%20Improvement.htm) and it was also withstood severe cold conditions in different test locations. Through this research work we could successfully develop an improved version of Tellahamsa by introgressing four major genes conferring resistance to two major diseases (bacterial blight and blast) through marker assisted backcross breeding coupled with stringent phenotypic selection.

## Conclusions

The current study was undertaken with a view to introgress resistance genes of BB (*Xa21 + xa13*) and blast (*Pi54 + Pi1*) into the background of cold tolerant mega rice variety Tellahamsa. Two improved ICF_3_ lines #TH-625-159 and #TH-625-491 possessing > 94% RPG along with BB and blast resistance was the outcome from the current study. In addition to the similarity of these lines to recurrent parent in different agronomic and quality traits. Thus, the developed pyramided lines can be used as a potential donor for BB and blast resistance in different future pre-breeding programs.

## Supporting information

S1 Fig Selection of ICF_2_ plants having *xa13*, *Xa21*, *Pi54 and Pi1* genes with foreground selection markers. (DOCX)

S2 Fig Blast nursery screening of the intercross (ICF_2_) progenies at Maruteru 3(a), Nellore 3 (b) and Hyderabad 3 (c), during *Rabi* 2014–15. Bacterial blight nursery screening of the intercross (ICF_2_) progenies at Hyderabad 3 (d) and Maruteru 3(e) during *Rabi* 2014–15.
(DOCX)

S3 Fig Selection of ICF_3_ (TH-625-159 and TH-625-491) plants/progenies having *xa13*, *Xa21*, *Pi54 and Pi1* genes. The foreground selection markers xa13 *prom*, pTA248, *Pi54-MAS* and RM224 were used for screening of *xa13*, *Xa21*, *Pi54 and Pi1* genes respectively, in the ICF_3_ plants through PCR. Gel (i), (ii), (iii), and (iv) represents all the ICF_3_ plants are “homozygous positive plants” with suitable BB and blast target genes. TH: Tellahamsa; B: B95-1/ISM; N: NLR145; 50bp: Ladder.
(DOCX)

S1 Table List of gene specific/ linked markers used for the identification of major BB and blast resistance genes. (DOCX)

S2 Table Agro-morphological parameters of selected ICF_2_ plants. (DOCX)

S3 Table Screening of selected intercross (ICF_2_) plants for their resistance against blast and BB disease during *Rabi*, 2014–15 season at different locations. (DOCX)

S1 Raw images (PDF)
