# Supplementary material for: Marker Assisted Gene Pyramiding (MAGP) for bacterial blight and blast resistance into mega rice variety “Tellahamsa”
Source: PLoS One. 2020 Jun 19;15(6):e0234088. doi: 10.1371/journal.pone.0234088 (PMC7304612; doi:10.1371/journal.pone.0234088)

**Supplementary Figure 1:** Selection of ICF_2_ plants having *xa13, Xa21, Pi54 and Pi1* genes with foreground selection markers.


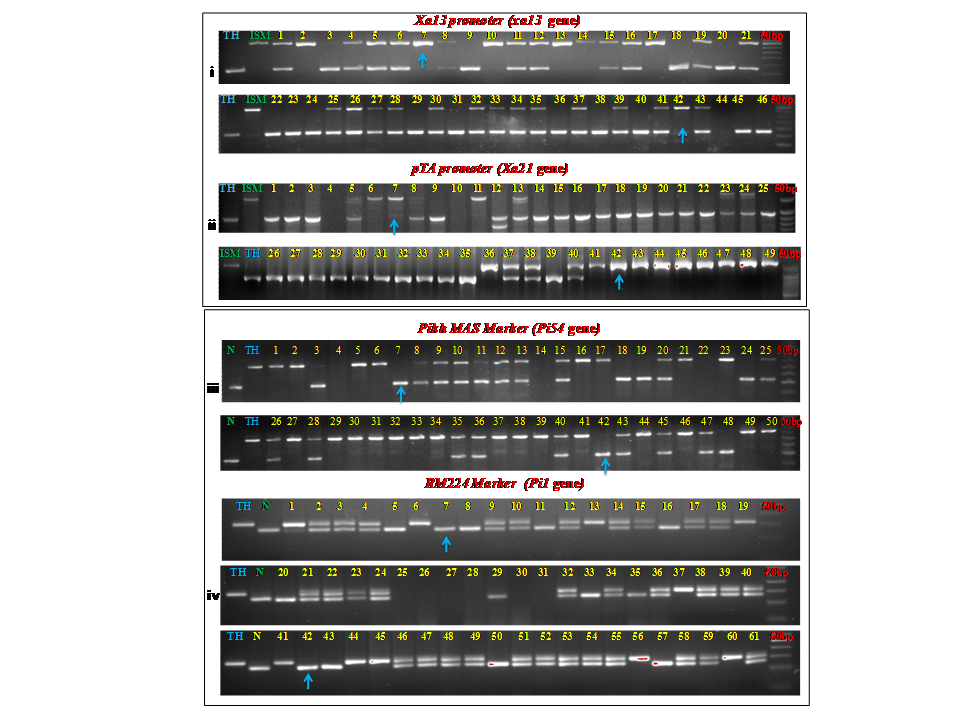

Supplement: S1 Fig — (DOCX) [file pone.0234088.s001.docx]
