# Supplementary material for: Marker Assisted Gene Pyramiding (MAGP) for bacterial blight and blast resistance into mega rice variety “Tellahamsa”
Source: PLoS One. 2020 Jun 19;15(6):e0234088. doi: 10.1371/journal.pone.0234088 (PMC7304612; doi:10.1371/journal.pone.0234088)

**Supplementary figure 2:** Blast nursery screening of the intercross (ICF_2_) progenies at Maruteru 3(a), Nellore 3 (b) and Hyderabad 3 (c), during *Rabi* 2014-15. Bacterial blight nursery screening of the intercross (ICF_2_) progenies at Hyderabad 3 (d) and Maruteru 3(e) during *Rabi* 2014-15


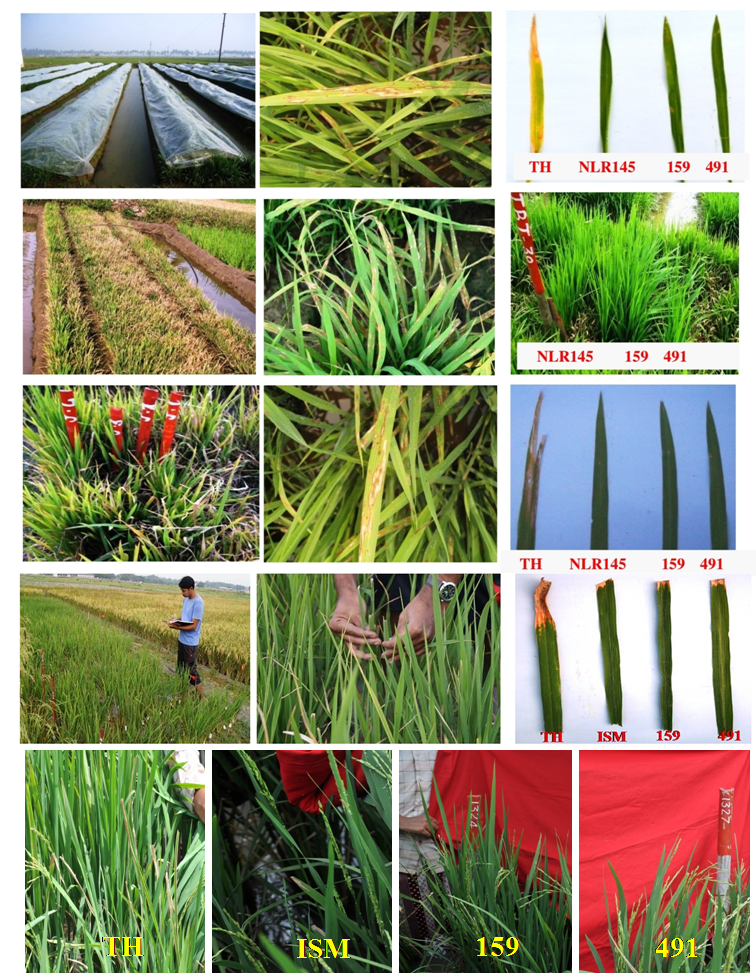

Supplement: S2 Fig — Blast nursery screening of the intercross (ICF2) progenies at Maruteru 3(a), Nellore 3 (b) and Hyderabad 3 (c), during Rabi 2014–15. Bacterial blight nursery screening of the intercross (ICF2) progenies at Hyderabad 3 (d) and Maruteru 3(e) during Rabi 2014–15. (DOCX) [file pone.0234088.s002.docx]
