# Supplementary material for: Marker Assisted Gene Pyramiding (MAGP) for bacterial blight and blast resistance into mega rice variety “Tellahamsa”
Source: PLoS One. 2020 Jun 19;15(6):e0234088. doi: 10.1371/journal.pone.0234088 (PMC7304612; doi:10.1371/journal.pone.0234088)

**Supplementary figure 3:** Selection of ICF_3_ (TH-625-159 and TH-625-491) plants/progenies having *xa13, Xa21, Pi54 and Pi1* genes. The foreground selection markers xa13 *prom*, pTA248, *Pi54-MAS* and RM224 were used for screening of *xa13, Xa21, Pi54 and Pi1* genes respectively, in the ICF_3_ plants through PCR. Gel (i), (ii), (iii), and (iv) represents all the ICF_3_ plants are “homozygous positive plants” with suitable BB and blast target genes. TH: Tellahamsa; B: B95-1/ISM; N: NLR145; 50bp: Ladder.


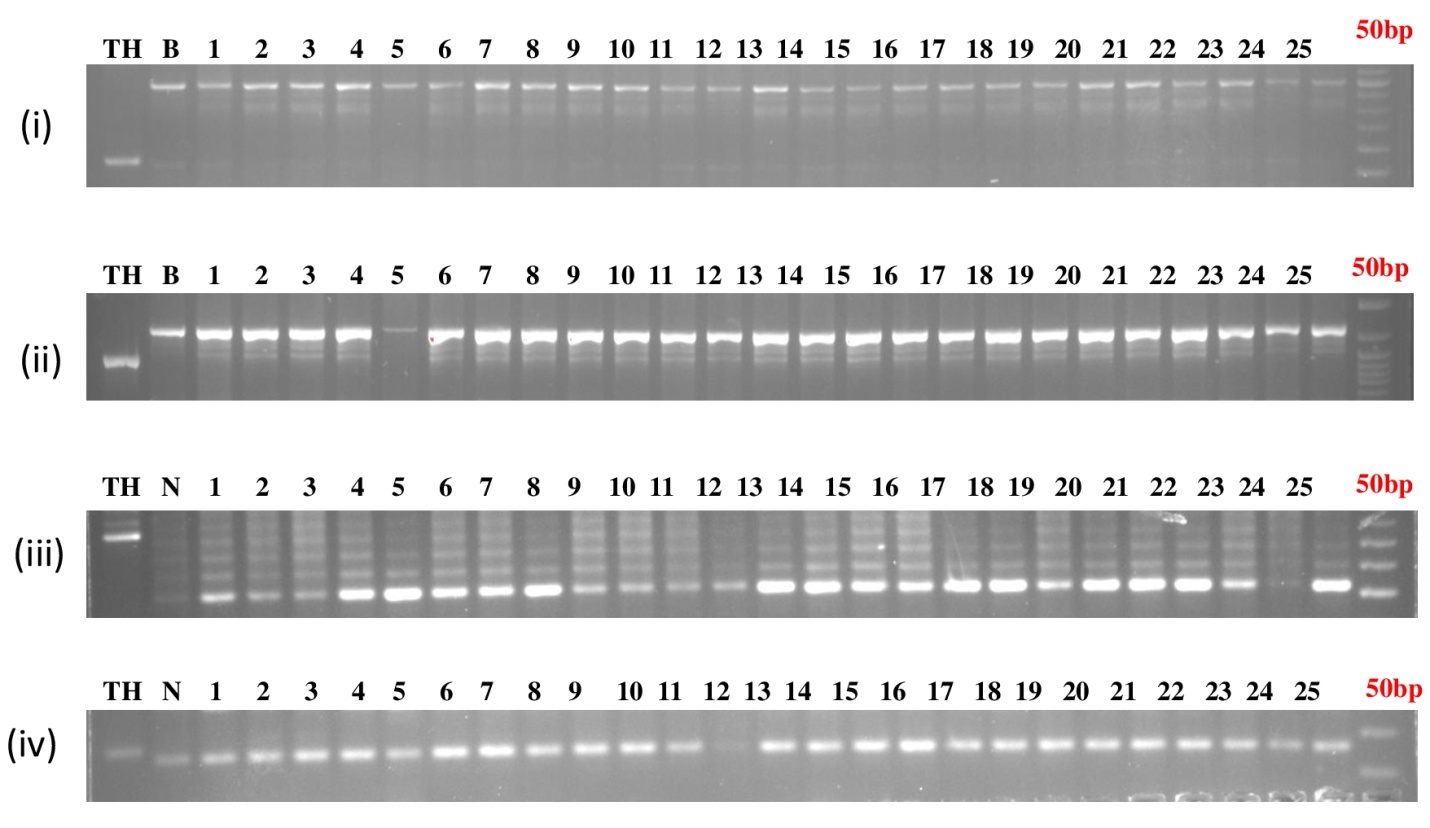

Supplement: S3 Fig — The foreground selection markers xa13 prom, pTA248, Pi54-MAS and RM224 were used for screening of xa13, Xa21, Pi54 and Pi1 genes respectively, in the ICF3 plants through PCR. Gel (i), (ii), (iii), and (iv) represents all the ICF3 plants are “homozygous positive plants” with suitable BB and blast target genes. TH: Tellahamsa; B: B95-1/ISM; N: NLR145; 50bp: Ladder. (DOCX) [file pone.0234088.s003.docx]
