# Supplementary material for: Marker Assisted Gene Pyramiding (MAGP) for bacterial blight and blast resistance into mega rice variety “Tellahamsa”
Source: PLoS One. 2020 Jun 19;15(6):e0234088. doi: 10.1371/journal.pone.0234088 (PMC7304612; doi:10.1371/journal.pone.0234088)
Supplement: S1 Table — (DOCX) [file pone.0234088.s004.docx]

**Supplementary Table 1**

**List of gene specific/ linked markers used for the identification of major BB and blast resistance genes**

| ***Gene*** | ***Primers*** | ***Resistance allele*** | ***Susceptible allele*** | ***Chr*** | ***References*** |
| --- | --- | --- | --- | --- | --- |
| *xa13* | *xa13 Promoter (xa13)- BLB* | 500bp | 250bp | 8 | *Sundaram et al.(2008)* |
| *Xa21* | *pTA248 (Xa21) - BLB* | 900bp | 700bp | 11 | *Ronold et al. (1992)* |
| *Pi54* | *Pi54 MAS*  *(Pi54) -Blast* | 210bp | 350bp | 11 | *Ram kumar et al. (2011)* |
| *Pi1* | *RM224 (Pi1)-Blast* | 130bp | 160bp | 11 | *Hittalmani et al. (2000)* |
