# Supplementary material for: Marker Assisted Gene Pyramiding (MAGP) for bacterial blight and blast resistance into mega rice variety “Tellahamsa”
Source: PLoS One. 2020 Jun 19;15(6):e0234088. doi: 10.1371/journal.pone.0234088 (PMC7304612; doi:10.1371/journal.pone.0234088)
Supplement: S2 Table — (DOCX) [file pone.0234088.s005.docx]

**Supplementary Table 2**

**Agro-morphological parameters of selected ICF_2_ plants**

PI = Plant identity; TG = Target genes; DFF = Days to 50% Flowering; PH = Plant height (cm); NPP = No. of productive panicles per plant; PL = Panicle length (cm); NGP = No. of grains per panicle; GYP = Grain yield per plant (g); 1000 SW = 1000 seed weight (g); GT = Grain type; GR % = Genome recovery in %

Agro morphological characters of selected ICF_2_four and three genes pyramid lines *(Xa21 + xa13 + Pi54 + Pi1, Xa21 + Pi54 + Pi1, Xa21 + xa13 + Pi54* and *Xa21 + xa13 + Pi1)* were collected at ARI, Hyderabad during Rabi, 2014-15

**#** Based on recurrent parent genome recovery and other phenotypic characters two plants ICF_2_-TH-625-159 and ICF_2_-TH-625-491 were selected.

| **S. No** | **Plant identity** | **Allelic status of *target genes*** | **Days to 50% Flowering** | **Plant height (cm)** | **No. of productive panicles per plant** | **Panicle length (cm)** | **No. of grains per panicle** | **Grain yield per plant (g)** | **Thousand seed weight (g)** | **Grain type** | **Genome recovery in %** |
| --- | --- | --- | --- | --- | --- | --- | --- | --- | --- | --- | --- |
|  | **Tellahamsa** | **-** | **90** | **95** | **14** | **26.5** | **130** | **40.5** | **23.5** | **LS** |  |
| **1** | **ICF_2_-TH-625- 21** | ***Xa21 + xa13 + Pi54 + Pi1*** | **98** | **98** | **12** | **24.5** | **120** | **27.4** | **21.5** | **LS** | **89.1%** |
| **2** | **ICF_2_-TH-625-105** | ***Xa21 + xa13 + Pi54*** | **105** | **101** | **10** | **25.1** | **124** | **22.0** | **21.8** | **LS** | **89.2%** |
| **3** | **ICF_2_-TH-625-159***^#^* | ***Xa21 + xa13 + Pi54 + Pi1*** | **97** | **101** | **14** | **26.5** | **140** | **43.5** | **25.0** | **LS** | **94.8%** |
| **4** | **ICF_2_-TH-625-211** | ***Xa21 + xa13 + Pi54*** | **101** | **100** | **13** | **25.5** | **105** | **27.3** | **20.0** | **LS** | **89.9%** |
| **5** | **ICF_2_-TH-625-325** | ***Xa21 + xa13 + Pi1*** | **106** | **98** | **14** | **26.2** | **108** | **24.4** | **19.5** | **LS** | **90.8%** |
| **6** | **ICF_2_-TH-625-491***^#^* | ***Xa21 + xa13 + Pi54 + Pi1*** | **90** | **96.5** | **13** | **26.2** | **135** | **41.7** | **23.2** | **LS** | **95.6%** |
| **7** | **ICF_2_-TH-625-501** | ***Xa21 + xa13 + Pi54 + Pi1*** | **98** | **98** | **9** | **24.7** | **114** | **29.1** | **18.7** | **LS** | **91.1%** |
| **8** | **ICF_2_-TH-625-588** | ***Xa21 + Pi54 + Pi1*** | **97** | **100** | **11** | **23** | **115** | **35.8** | **21.5** | **LS** | **91.2%** |
| **9** | **ICF_2_-TH-625-624** | ***Xa21 + xa13 + Pi54*** | **99** | **98.5** | **8** | **24.8** | **104** | **17.8** | **20.5** | **LS** | **92.5%** |
