# Supplementary material for: Marker Assisted Gene Pyramiding (MAGP) for bacterial blight and blast resistance into mega rice variety “Tellahamsa”
Source: PLoS One. 2020 Jun 19;15(6):e0234088. doi: 10.1371/journal.pone.0234088 (PMC7304612; doi:10.1371/journal.pone.0234088)
Supplement: S3 Table — (DOCX) [file pone.0234088.s006.docx]

**Supplementary Table 3**

**Screening of selected intercross (ICF_2_) plants for their resistance against blast and BB disease during *Rabi*, 2014-15 season at different locations**

| S. No | Plant identity | Allelic status of *Xa21 + xa13 + Pi54 + Pi1* | Blast disease score at Maruteru (IB-16) | Blast disease score at Nellore (ID-14) | Blast disease  score at Hyderabad (NLR-1) | BB disease score at Maruteru  (IC-31) | BB disease score at Hyderabad (DX-020) |
| --- | --- | --- | --- | --- | --- | --- | --- |
|  | Tellahamsa |  | 9 | 9 | 8 | 9 | 9 |
|  | NLR145 |  | 1 | 2 | 1 | - | - |
|  | ISM |  |  |  |  | 1 | 1 |
| 1 | ICF_2_-TH-625- 21 | *Xa21 + xa13 + Pi54 + Pi1* | 3 | 5 | 4 | 3 | 5 |
| 2 | ICF_2_-TH-625-105 | *Xa21 + xa13 + Pi54* | 2 | 5 | 5 | 3 | 1 |
| 3 | ICF_2_-TH-625-159 | *Xa21 + xa13 + Pi54 + Pi1* | 1 | 1 | 1 | 1 | 1 |
| 4 | ICF_2_-TH-625-211 | *Xa21 + xa13 + Pi54* | 6 | 8 | 6 | 5 | 3 |
| 5 | ICF_2_-TH-625-325 | *Xa21 + xa13 + Pi1* | 3 | 5 | 4 | 5 | 3 |
| 6 | ICF_2_-TH-625-491 | *Xa21 + xa13 + Pi54 + Pi1* | 2 | 1 | 2 | 1 | 1 |
| 7 | ICF_2_-TH-625-501 | *Xa21 + xa13 + Pi54 + Pi1* | 5 | 8 | 8 | 3 | 1 |
| 8 | ICF_2_-TH-625-588 | *Xa21 + Pi54 + Pi1* | 3 | 5 | 8 | 5 | 3 |
| 9 | ICF_2_-TH-625-624 | *Xa21 + xa13 + Pi54* | 8 | 9 | 6 | 5 | 3 |

Four and three genes pyramid lines at ICF_2_ generation plants *(Xa21 + xa13 + Pi54 + Pi1, Xa21 + Pi54 + Pi1, Xa21 + xa13 + Pi54 and Xa21 + xa13 + Pi1)* were screened with local blast isolates of three different locations (West Godavari, Nellore and Hyderabad) and two different locations with bacterial blight isolates (West Godavari and Hyderabad) under controlled conditions by UBN method.
