# Supplementary material for: Marker Assisted Gene Pyramiding (MAGP) for bacterial blight and blast resistance into mega rice variety “Tellahamsa”
Source: PLoS One. 2020 Jun 19;15(6):e0234088. doi: 10.1371/journal.pone.0234088 (PMC7304612; doi:10.1371/journal.pone.0234088)

Original uncropped and unadjusted gel images for supplementary figure 1

***Xa13 promoter (xa13* gene)**

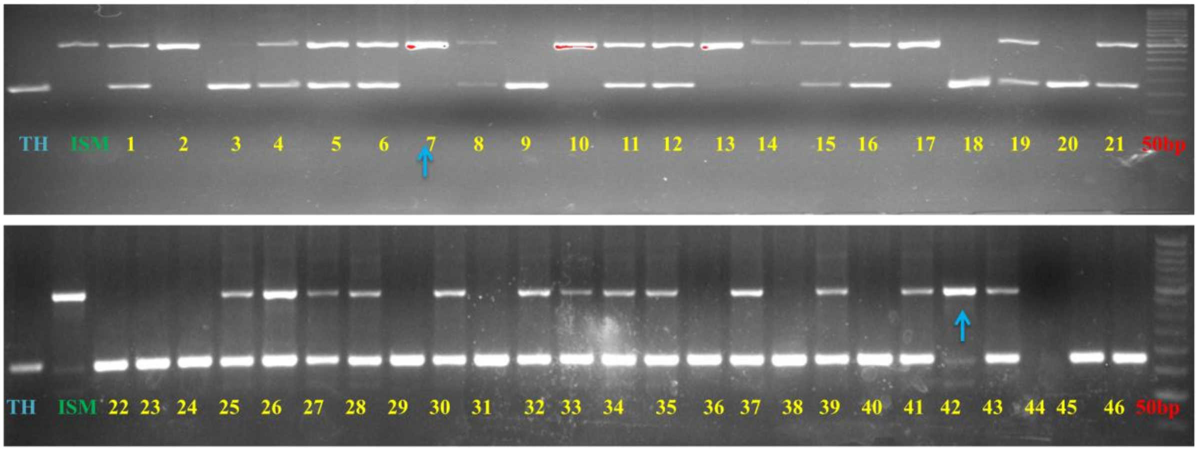

***pTA 248 (Xa21* gene)**

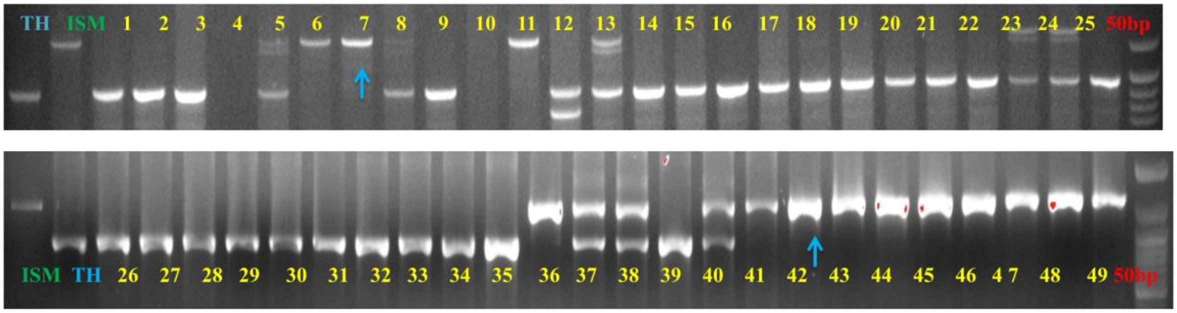

Pi54 MAS (Pi54 gene)

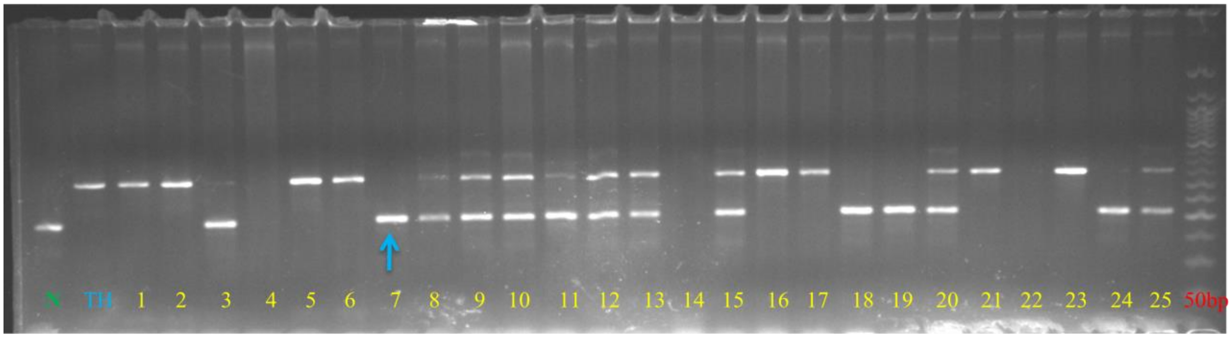

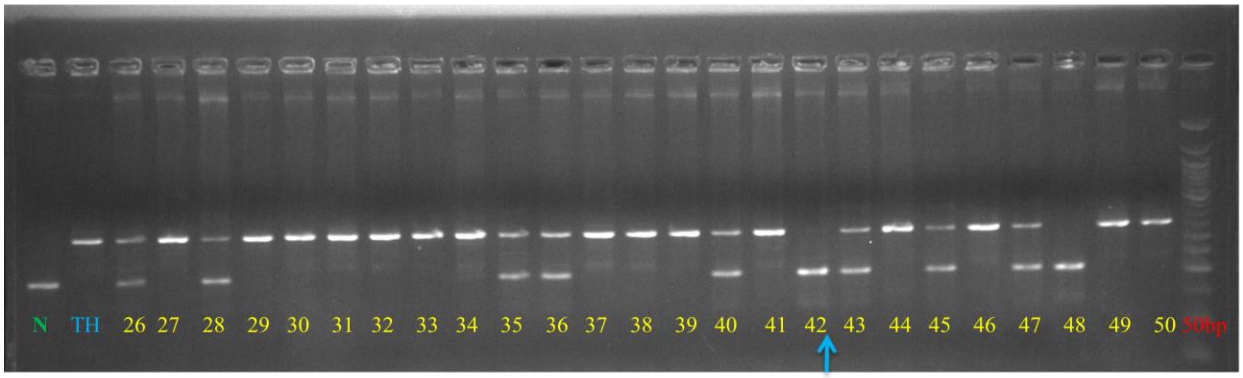

RM224 (Pi1 **gene)**

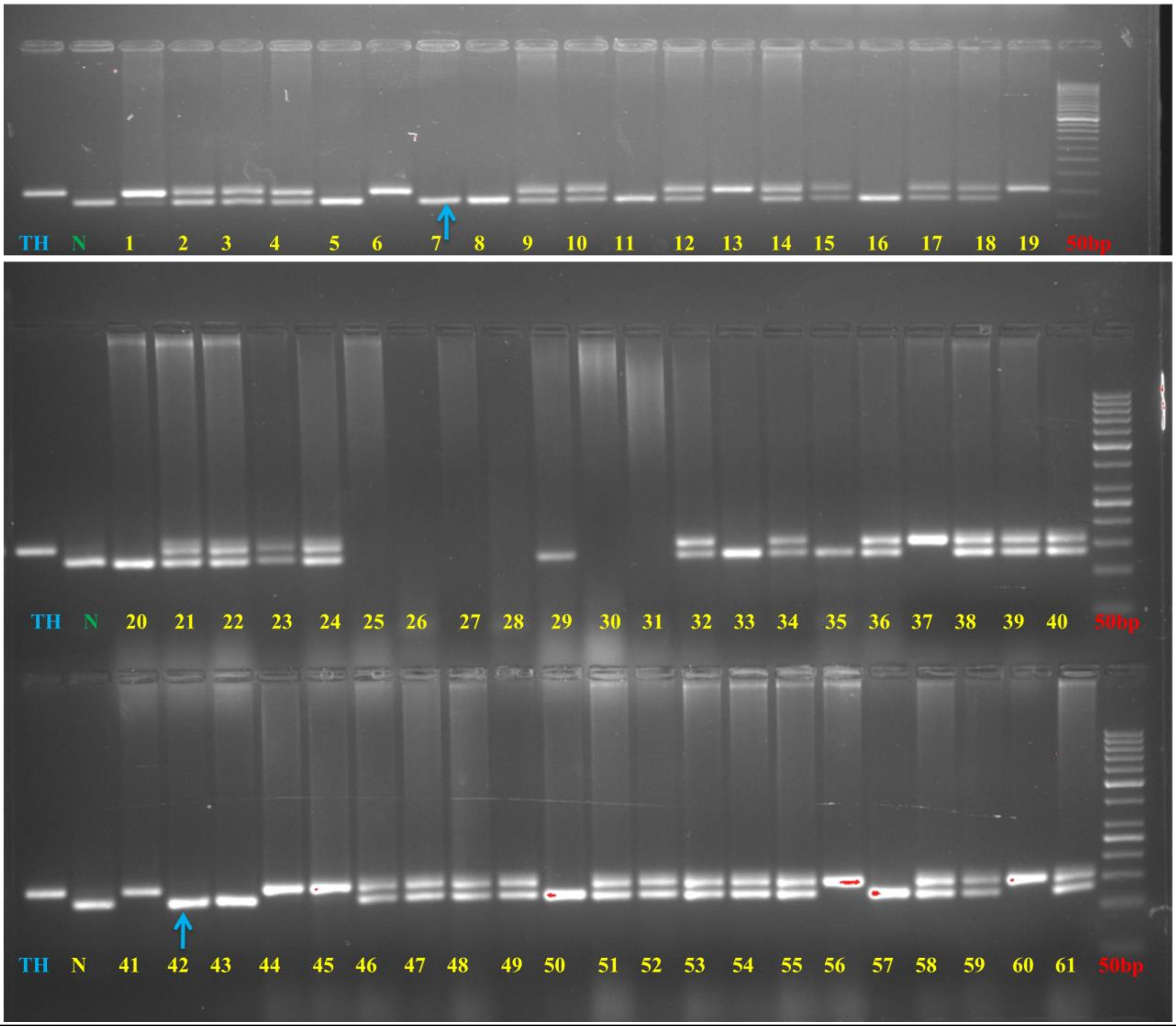

Original uncropped and unadjusted gel images for <u>supplementary figure 3</u>

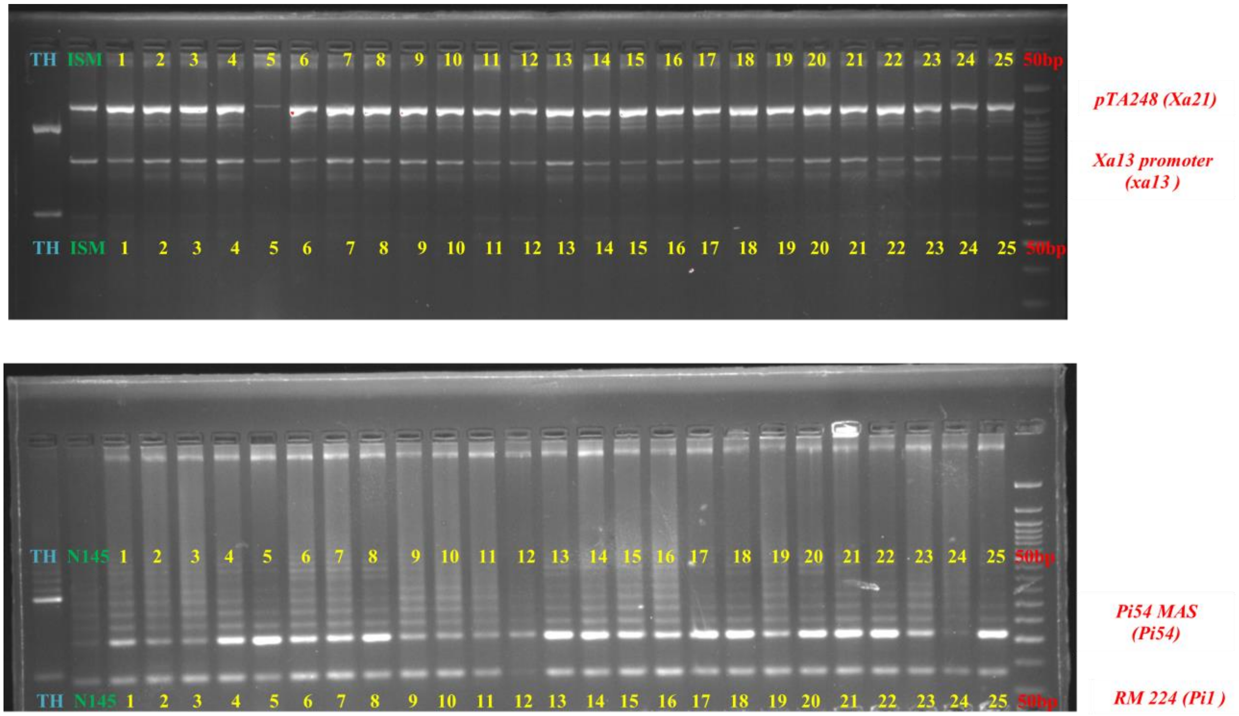

Supplement: S1 Raw images — (PDF) [file pone.0234088.s007.PDF]
